# Co-crystalization reveals the interaction between AtYchF1 and ppGpp

**DOI:** 10.3389/fmolb.2022.1061350

**Published:** 2022-11-30

**Authors:** Ming-Yan Cheung, Xiaorong Li, Yee-Shan Ku, Zhongzhou Chen, Hon-Ming Lam

**Affiliations:** ^1^ Center for Soybean Research of the State Key Laboratory of Agrobiotechnology and School of Life Sciences, The Chinese University of Hong Kong, Shatin, Hong Kong SAR, China; ^2^ State Key Laboratory of Agrobiotechnology, College of Biological Sciences, China Agricultural University, Beijing, China

**Keywords:** Arabidopsis, protein crystallography, G-protein, GTPase, ppGpp, RelA/SpoT homolog, stress response, YchF

## Abstract

AtYchF1 is an unconventional G-protein in *Arabidopsis thaliana* that exhibits relaxed nucleotide-binding specificity. The bindings between AtYchF1 and biomolecules including GTP, ATP, and 26S rRNA have been reported. In this study, we demonstrated the binding of AtYchF1 to ppGpp in addition to the above molecules. AtYchF1 is a cytosolic protein previously reported as a negative regulator of both biotic and abiotic stresses while the accumulation of ppGpp in the cytoplasm induces retarded plant growth and development. By co-crystallization, *in vitro* pull-down experiments, and hydrolytic biochemical assays, we demonstrated the binding and hydrolysis of ppGpp by AtYchF1. ppGpp inhibits the binding of AtYchF1 to ATP, GTP, and 26S rRNA. The ppGpp hydrolyzing activity of AtYchF1 failed to be activated by AtGAP1. The AtYchF1-ppGpp co-crystal structure suggests that ppGpp might prevent His^136^ from executing nucleotide hydrolysis. In addition, upon the binding of ppGpp, the conformation between the TGS and helical domains of AtYchF1 changes. Such structural changes probably influence the binding between AtYchF1 and other molecules such as 26S rRNA. Since YchF proteins are conserved among different kingdoms of life, the findings advance the knowledge on the role of AtYchF1 in regulating nucleotide signaling as well as hint at the possible involvement of YchF proteins in regulating ppGpp level in other species.

## 1 Introduction

G-proteins refer to proteins which could bind GTP (Saraste et al., 1990). Some G-proteins possess GTPase activities (Saraste et al., 1990). Although not all G-proteins harbor a phosphate-binding loop (P-loop), P-loop has been known as a common binding site for ATP and GTP (Saraste et al., 1990). Based on the conserved amino acid sequences and structural features, G proteins are generally categorized into two classes, TRAFAC (translation factors) and SIMIBI (signal recognition, MinD, BioD) (Mittenhuber, 2001; Leipe et al., 2002). TRAFAC proteins are mostly involved in the regulation of translation, signal transduction and intracellular transport while SIMIBI proteins are mostly involved in the regulation of protein localization, chromosome partitioning, membrane transport and metabolic enzymatic activities (Mittenhuber, 2001; Leipe et al., 2002). YchF1 is a P-loop TRAFAC GTPases belonging to the Obg family under the superfamily Obg-Hflx (Suwastika et al., 2014).

Proteins belonging to the Obg family are ancient unconventional G-proteins (Cheung et al., 2010). YchF proteins are highly conserved among different kingdoms of living organisms (Cheung et al., 2010). However, the exact functions of YchF proteins have remained elusive. YchF proteins are uniquely featured by the relaxed nucleotide binding specificity to bind both ATP and GTP. We previously revealed the crystal structures of rice YchF1 protein (OsYchF1) in ATP-bound, GTP-bound and apo forms (Cheung et al., 2016). OsYchF1 harbors a G domain at the N-terminus for GTP and ATP binding and hydrolysis, and a TGS (ThrRS, GTPase, and SpoT) domain in the C-terminus for nucleic acid binding (Cheung et al., 2016). 26S rRNA was found to bind with OsYchF1 at the TGS domain (Cheung et al., 2016). Within G domain, there are five motifs (G1 to G5). G1 motif (NVGKST) recognizes the phosphate group of ATP and GTP. G2 (*X*TI) and G3 (hhhDIAG) motifs are involved in the coordination of a Mg^2+^ ion that is required for nucleotide binding and hydrolysis. G4 motif (NMSE) (different from canonical G4 ((N/T) KXD) exhibits relaxed specificity in nucleotide binding. G5 motif (SCA) supports guanine base recognition (Koller-Eichhorn et al., 2007; Cheung et al., 2016). A helical domain is inserted between G3 and G4 motif (Cheung et al., 2016). The helical domain was suggested to be a modulator of the G protein to interact with its effector.

In prokaryotes, several reports have suggested that Obg family members are associated with ribosomal subunits and participate in translation. For example, in *Escherichia coli*, the Era protein was found to be associated with 16S rRNA and the 30S ribosomal subunit (Sayed et al., 1999). The depletion of Era in *E. coli* resulted in translational defects *in vivo* and *in vitro* (Sayed et al., 1999). The CgtAE protein in *E. coli* was found to be co-fractionated with the 50S ribosomal subunit (Wout et al., 2004). In addition, CgtAE was found to interact with SpoT (ppGpp synthetase/hydrolase) which is involved in stringent responses under stress conditions (Wout et al., 2004). The structure of the Obg protein in *Bacillus subtilis*, BsObg, was revealed by crystallization in a nucleotide-bound form (Buglino et al., 2002). It was suggested that the nucleotide-bound configuration was probably a result of co-purification from the lysate of *E. coli.,* in which BsObg was expressed ectopically (Buglino et al., 2002). Upon careful review of the crystal structure, the electron density within the P loop was postulated as ppGpp, instead of GDP and two magnesium ions as first expected, due to the ligand having a bigger molecular size than GDP, with additional atoms emanating from the 3′-OH group of ribose. ppGpp was the only nucleotide which is able to accumulate up to the millimolar level under stress conditions in bacteria (Buglino et al., 2002).

In eukaryotes, the yeast NOG1 protein, which belongs to the Obg subfamily, was found to co-precipitate with free 60S ribosomes and localized in the nucleolus. The mutation of NOG1 in yeast was lethal, while the conditional mutation of NOG1 resulted in a reduced level of the 60S ribosomal subunit. NOG1 was therefore suggested as an essential protein involved in 60S ribosome biogenesis (Kallstrom et al., 2003). In the protozoan *Trypanosoma cruzi*, when cell lysates were fractionated on a sucrose gradient, TcYchF was detected in the fractions corresponding to 40S, 60S and 80S ribosomes and polysomes (Gradia et al., 2009). Upon treatment with puromycin for polysome disassembly, TcYchF was found to be dissociated from ribosomes committed to translation (Gradia et al., 2009). The interactions between TcYchF and ribosomal subunits, including 40S S7e and 60S L26, were further confirmed by co-immunoprecipitation experiments (Gradia et al., 2009). The YchF protein from rice (OsYchF1) was previously characterized and a specific interaction between its TGS domain and the 26S rRNA of rice was identified (Cheung et al., 2010).

AtYchF1 *in Arabidopsis thaliana* is a close homolog of OsYchF1, which possesses a noncanonical G4 sequence (NxxE) within the G domain, instead of the canonical NKxD in most P-loop GTPases, resulting in a relaxed nucleotide-binding specificity and the ability to hydrolyze both ATP and GTP (Cheung et al., 2016). In contrast, the human homolog of OsYchF1, hOLA1, can only bind ATP (Koller-Eichhorn et al., 2007). Moreover, OsYchF1 was characterized as a negative regulator of plant biotic (Cheung et al., 2010) and abiotic (Cheung et al., 2013) stress responses. Upon salt treatment, transgenic Arabidopsis ectopically expressing *OsYchF1* had more severe leaf chlorosis, reduced chlorophyll contents, and higher levels of ion leakage and lipid peroxidation (Cheung et al., 2013). *OsYchF1*-overexpressing Arabidopsis plants were more susceptible to infection by *Pseudomonas syringae* pv. *tomato* strain DC3000 (*Pst* DC3000) (Cheung et al., 2016). However, by substituting the ectopically expressed native OsYchF1 with a mutant version of OsYchF1 having a mutated G4 motif to restrict its ligand-binding specificity to GTP-only, the disease-susceptible phenotype was abolished in the transgenic Arabidopsis (Cheung et al., 2016). The negative regulatory role of OsYchF1 in disease resistance was therefore dependent on its ATP-binding capacity (Cheung et al., 2016). The findings highlight the roles of specific nucleotides in regulating the function of OsYchF1.

The amino acid sequences of OsYchF1 and AtYchF1 are highly conserved (Cheung et al., 2010). Similar to OsYchF1, AtYchF1 was also previously characterized as negative regulator for both biotic and abiotic stresses (Cheung et al., 2010; Cheung et al., 2013; Cheung et al., 2016). OsYchF1 and AtYchF1 are active when being bound with ATP and GTP (Cheung et al., 2010; Cheung et al., 2013; Cheung et al., 2016). When being bound with 26S rRNA, OsYchF1 and AtYchF1, which are TRAFAC GTPases, were suggested to be possible regulators of the translation machinery (Cheung et al., 2010). In addition, by microscopic study and protein co-crystallization, OsYchF1 was shown to interact with OsGAP1 (*Oryza sativa* GTPase Activating Protein 1) (Cheung et al., 2010; Cheung et al., 2016). Under normal condition, OsYchF1 and OsGAP1 localize in the cytosol (Cheung et al., 2010). However, upon wounding, both OsYchF1 and OsGAP1 re-localize to the plasma membrane (Cheung et al., 2010). The phospholipid binding property of OsGAP1 was later demonstrated in another report (Yung et al., 2015). It was therefore suggested that, upon wounding, OsYchF1 re-localized to the plasma membrane due to its interaction with OsGAP1 (Cheung et al., 2010; Yung et al., 2015). Biochemical assays showed that OsGAP1 activates both the ATP and GTP hydrolytic activity of OsYchF1 and AtYchF1 (Cheung et al., 2010; Cheung et al., 2013). Being opposite to OsYchF1 and AtYchF1, *in planta* data suggested that OsGAP1 and AtGAP1 are positive regulators for biotic and abiotic stresses (Cheung et al., 2008; Cheung et al., 2013; Yung et al., 2015). In addition, OsGAP1 and AtGAP1 were also shown to compete with 26S rRNA for the binding to OsYchF1 (Cheung et al., 2010). The binding by OsGAP1 and AtGAP1 was suggested to be a mechanism for inhibiting the translational regulatory function of OsYchF1 (Cheung et al., 2010).

While ATP and GTP have been reported to regulate various biological processes (Wittinghofer, 2016), ppGpp has been known as an alarmone that mediates stringent responses to stress (Maekawa et al., 2015; Fernández-Coll and Cashel, 2020; Ito et al., 2020). The ppGpp-mediated stringent response has been extensively studied in bacteria. Under stress conditions, such as nutrient starvation, ribosome-associated proteins belonging to the RelA/SpoT superfamily mediate the synthesis of ppGpp (Diez et al., 2020; Fernández-Coll and Cashel, 2020). The abrupt increase in the level of ppGpp trigger the bacteria to enter metabolic quiescence (Diez et al., 2020; Fernández-Coll and Cashel, 2020). Upon an increase in the cellular ppGpp level, the proteome is reprogrammed due to the halt in DNA, RNA and protein synthesis, until favorable growth conditions resume (Vinogradova et al., 2020). ppGpp binds to translational GTPases, such as IF2, to inhibit translation initiation, thus reducing the rate of protein synthesis (Vinogradova et al., 2020). In bacteria, numerous proteins are found to exhibit (p)ppGpp synthetase and/or hydrolase activities. The largest group among these proteins is characterized as RelA/SpoT Homolog (RSH) enzymes. Most of the RSH enzymes exhibit both synthetase and hydrolase activities. The C-terminus of the protein plays a regulatory role while the N-terminus possesses the catalytic domain. Upon sensing the levels of molecules such as fatty acids, carbon sources and cyclic di-AMP, the conformation of the catalytic site is altered by the regulatory domain to bring about the synthetase or hydrolase activity (Peterson et al., 2020).

In plants, the synthesis and hydrolysis of ppGpp are both reported to be achieved by RSH proteins, which are encoded by the nuclear genome (Boniecka et al., 2017). However, plant RSH proteins possess a chloroplastic transit peptide which mediates the localization of the protein to the chloroplast, where ppGpp is synthesized or hydrolyzed depending on the stress conditions (Boniecka et al., 2017). Under salt stress, the expressions of *RSH* genes are induced (Field, 2018). The increased ppGpp level leads to reduced rates of photosynthesis and cell division. In such a quiescence-like state, plant growth is inhibited but the survival is maintained (Avilan et al., 2021). In plant, under abiotic and biotic stresses, the *in planta* ppGpp level was found to be induced (Takahashi et al., 2004). Although the transit peptide mediates the chloroplastic localization of the RSH proteins in plants, cytoplasmic localization of RSH proteins in plants is also possible. In Arabidopsis, RSH1 was found to be an interacting partner of a cytoplasmic disease-resistance protein RPP5 (van der Biezen et al., 2000). Therefore, AtRSH1 was predicted as a membrane-anchored cytoplasmic molecule with significant homology to bacterial RelA and SpoT proteins (van der Biezen et al., 2000). ppGpp has also been detected in animal cells including *Drosophila* and human cells, which do not contain chloroplast (Ito et al., 2020). In *Drosophila*, ppGpp-mediated stringent responses were reported (Ito et al., 2020). The accumulation of ppGpp induced metabolic changes, cell death, and lethality (Ito et al., 2020).

Inspired by the relaxed nucleotide-binding specificity of OsYchF1 (Cheung et al., 2016), we hypothesized a similarly relaxed nucleotide-binding specificity of AtYchF1. By biochemical assays, we characterized the binding and hydrolysis of ppGpp, besides GTP and ATP, by AtYchF1. The ppGpp binding and hydrolytic activities of AtYchF1 were further analyzed by the co-crystal structure of AtYchF1-ppGpp. Co-crystallization data suggested that the binding of ppGpp to AtYchF1 inhibits the binding between AtYchF1 and other molecules including ATP, GTP, and 26S rRNA. The findings advance the knowledge on the role of AtYchF1 in regulating nucleotide sigaling. Since YchF proteins are conserved among different kingdoms of organisms including prokaryotes and animals, the type of interaction between AtYchF1 and ppGpp could also potentially occur in other YchF homologs in their regulation of stringent response.

## 2 Materials and methods

### 2.1 Gene cloning

Gene constructs in this report were amplified by PCR from cDNAs generated by reverse transcription from total RNA, following the procedures in a previous report (Cheung et al., 2008). Specifically, *AtGAP1* and *AtYchF1* were amplified using PrimeSTAR GXL polymerase (Cat# R050B, Takara Bio, CA, United States) with the following PCR cycle: 94°C for 5 min; 30 cycles of 94°C for 30 s, 55°C for 30 s, 72°C for 1 min 30 s, and a final polymerization step at 72°C for 10 min. PCR products were purified and digested with corresponding restriction enzymes (Supplementary Table S1). For protein expression in bacterial cells, the digested products were purified and ligated with *EcoR*I-and*-Sal*I double*-*digested pMAL-C2 vector (New England Biolabs Inc., Beverly, MA, United States), *EcoR*I-and*-Sal*I double digested pGex-4T-1 vector (GE28-9545-49, GE Healthcare, Chalfont St Giles, England) or *Age*I-and-*Kpn*I double-digested pRSETA-HisSUMO vector (an in-house expression plasmid based on pRSET-A [Cat# V35120, Invitrogen, Carlsbad, CA, United States] with an N-terminal His-SUMO tag). All ligated products were transformed into DH5α competent cells and selected on Luria–Bertani (LB) plates containing 100 mg L^−1^ ampicillin for positive transformants. The positive clones were verified by colony PCR and Sanger sequencing. Detailed primer information for cloning is given in Supplementary Table S1.

### 2.2 Expression and purification of MBP- or GST-tagged recombinant proteins for nucleotide binding and hydrolysis assays

The plasmids pMAL-C2-*AtYchF1* and pGex-4T-1-*AtGAP1* and their corresponding empty-vector control plasmids (pMAL-C2: New England Biolabs Inc., Beverly, MA, United States; pGex-4T-1: GE28-9545-49, GE Healthcare, Chalfont St Giles, England) were transformed into the *E. coli* BL21 (DE3) strain. Protein expression was induced by 0.5 mM isopropyl β-D-thiogalactopyranoside (IPTG) in the growth medium (LB) with 100 mg L^−1^ ampicillin with shaking at 20°C overnight. SpinClean^TM^ MBP Excellose^®^ spin kit (Cat**#** 23020, Mbiotech, Haman, Korea) and MagneGST™ Protein Purification System (Cat# V8600, Promega, CA, United States) were then used to purify the expressed MBP- and GST-tagged proteins according to the user manuals, respectively.

### 2.3 Nucleotide binding assays

Nucleotide binding assays were performed as described (Rohn et al., 1999) using MBP-AtYchF1. Thirty-one mircomolar 2'/3′-O-(N-Methyl-anthraniloyl) (Mant)-GTP (Cat# M12415, Thermo Fisher Scientific, Waltham, MA, United States) or Mant-ATP (Cat# M12417, Thermo Fisher Scientific, Waltham, MA, United States) was mixed with about 100 μM MBP-AtYchF1 or MBP-only in each 160-μL reaction. For the competition assays between ppGpp and GTP/ATP for AtYchF1 binding, the Mant-GTP/-ATP:ppGpp molar ratios of 1:1, 1:2 and 1:3 were tested.

### 2.4 Protein expression and purification of AtYchF1 for complex formation

The AtYchF1 cDNA fragment was sub-cloned into an in-house HisSUMO vector for high-level expression in *E. coli* BL21 (DE3). Target protein expression, extraction, and purification in the bacterial system were performed as described previously (Cheung et al., 2016). Briefly, the protein was purified on a GeneScript High Affinity Ni-NTA Resin column and 300 mM imidazole in the lysis buffer was used to elute the target protein. The HisSUMO tag was removed with SUMO protease (Fong et al., 2011). Following cleavage of the HisSUMO tag, the dialyzed protein was again passed over a Ni-NTA column to remove the uncleaved AtYchF1 and HisSUMO tag. The untagged AtYchF1 was further purified *via* Superdex 200 10/300 gel filtration (Cat# 28990944, GE Healthcare, Chalfont St Giles, England) equilibrated in 20 mM Tris (pH 6.7), 150 mM KCl, and 1 mM dithiothreitol (DTT). The peak corresponding to AtYchF1 in the elution was collected and concentrated.

### 2.5 X-ray crystallography of the AtYchF1-ppGpp complex

Crystallizations were performed at 4 °C by the hanging-drop vapor diffusion method. Crystals for the AtYchF1-ppGpp complex were obtained by first mixing purified AtYchF1 (15 mg ml^−1^) with 1 mM ppGpp. AtYchF1-ppGpp crystals were grown in a buffer containing 18% PEG 3350, 0.1 M Tris (pH 8.67) and 0.02 M MgCl_2_. Crystals were cryoprotected in mother liquor containing 20% (v/v) ethylene glycol. Diffraction data were collected at the beamline BL17B of the Shanghai Synchrotron Radiation Facility. Data were indexed, integrated, and scaled using HKL 2000 (Otwinowski and Minor, 1997). The initial phases were found by molecular replacement with PHASER using the structure of OsYchF1 (PDB: 5EE0) (Cheung et al., 2016) as the search template. The model was then further refined by iterative cycles of automated and manual model-building using REFMAC5 (Murshudov et al., 1997) and COOT (Emsley et al., 2010). The model was first built manually in the program COOT, and refinement was carried out with REFMAC5. Data collection and processing statistics are shown in Supplementary Table S2. The atomic coordinates and structure factors for the AtYchF1-ppGpp crystal structure have been deposited with the Protein Data bank under the accession number 7Y9I.

### 2.6 Nucleotide hydrolysis assays

MBP-AtYchF1 or MBP-only protein was purified from *E. coli* as mentioned above for the nucleotide hydrolysis assays. GTP and ATP hydrolysis were tested using the EnzChek™ Phosphate Assay Kit (Cat# E6646, Thermo Fisher Scientific, Waltham, MA, United States) (Webb, 1992), while ppGpp hydrolysis was tested using the EnzChek™ Pyrophosphate Assay Kit (Cat# E6645, Thermo Fisher Scientific, Waltham, MA, United States). For the ppGpp hydrolysis assay, MBP-AtYchF1 or MBP protein at different concentrations was used with 1 mM ppGpp as the substrate. The end-point signal at 360 nm was detected in the reaction mixture after a 30-min incubation at room temperature as recommended by the manufacturer’s protocol. Afterwards, rates of nucleotide hydrolyses were determined with 300 μM MBP-AtYchF1 and ATP, GTP or ppGpp at different concentrations. All signals were normalized with signals from the corresponding protein-free reaction mixtures. The MBP protein was employed as the negative control. To determine the activation of the hydrolytic activity of AtYchF1 by AtGAP1, GST-AtGAP1 purified from *E. coli* using the MagneGST™ Protein Purification System (Cat# V8600, Promega, CA, United States) was added to the reaction mixture at a 100 μM final concentration. All hydrolyzed nucleotide signals were detected at 360 nm using the spectrometer Synergy H1 hybrid multi-mode reader (Agilent Technologies, CA, United States).

### 2.7 *In vitro* pull-down experiments of ppGpp-charged GST-AtYchF1 with MBP-AtGAP1

GST-AtYchF1 and GST-only proteins were expressed and purified from *E. coli* as mentioned above. The proteins were bound on MagneGST™ Particles and washed using the MagneGST^TM^ Protein Purification System (Cat# V8600, Promega, CA, United States) following the manufacturer’s protocol. After washing, the MagneGST™ Particle-bound GST-AtYchF1 or GST-only protein was charged with 2 mM ppGpp as described previously (Spangler et al., 2009). The ppGpp-charged protein bound to the MagneGST™ Particles were split into two equal aliquots for the *in vitro* pull-down experiment with MBP-AtGAP1 or MBP-only. The charging of ppGpp and the *in vitro* pull-down experiments were carried out at 4°C. The pull-down products were eluted by 50 mM reduced-glutathione solution (50 mM Tris-HCl [pH 8.1]) before being subjected to SDS-PAGE with the use of 12.5% acrylamide gel for separation. After that, the proteins were detected by western blot using anti-GST (Cat# G7781, Sigma-Aldrich, St Louis, MO, United States) and anti-MBP (Cat# M6295, Sigma-Aldrich, St Louis, MO, United States) antibodies, followed by horseradish peroxidase (HRP)-conjugated anti-rabbit (Cat# NA934V, Amersham Biosciences, Bath, United Kingdom) and HRP-conjugated anti-mouse (Cat# 172-1011, Bio-Rad, Hercules, CA, United States) secondary antibodies, respectively. GST-only and MBP-only were used as the respective negative controls. Clarity^TM^ Western ECL substrate (Cat# 170-5060, Bio-Rad, Hercules, CA, United States) and ChemiDoc MP Imaging instrument (Cat# 170-8381, Bio-Rad, Hercules, CA, United States) were used for signal detection.

### 2.8 ppGpp-charged GST-AtYchF1 and digoxigenin (DIG)-labeled 26S rRNA binding assays

Detailed information of the 26S rRNA cloning and the generation procedures of the digoxigenin (DIG)-labeled 26S rRNA species were described in a previous study (Cheung et al., 2010). The binding assays of 26S rRNA by ppGpp-charged GST-AtYchF1 or GST-only were similar to a previous study with minor modifications (Cheung et al., 2010). In brief, before incubating with DIG-labeled 26S rRNA, the MagneGST™ Particle-bound (Cat# V8600, Promega, CA, United States) GST-AtYchF1 or GST-only was charged with 0, 1, 2.5 or 5 mM ppGpp overnight at 4°C (Spangler et al., 2009). One microgram each of DIG-labeled 26S rRNA was then mixed with the various ppGpp-charged GST-AtYchF1 or ppGpp-charged GST-only reaction mixtures for 1 h at 4°C. After three washes with the nucleotide charging solution, the *in vitro* pull-down products were eluted with 50 mM reduced-glutathione solution (50 mM Tris-HCl [pH 8.1]) and blotted onto a nylon membrane (Cat# 11417240001, Roche, Basel, Switzerland) using a slot blot apparatus (Cat# 170-3938, Bio-Rad, Hercules, CA, United States) for detection. Anti-Digoxigenin-AP Fab fragments (Cat# 11093274910, Roche, Basel, Switzerland) and anti-rabbit IgG F (ab’)_2_ fragment-alkaline phosphatase antibody (Cat# A0418, Sigma-Aldrich, St Louis, MO, United States) were used to detect DIG-labeled 26S rRNA and GST-AtYchF1 or GST-only protein loading, respectively. SIGMAFAST^TM^ BCIP^®^/NBT tablet (Cat# B5655-25TAB, Sigma-Aldrich, St Louis, MO, United States) was used as the substrate for detection. ChemiDoc MP Imaging instrument (Cat# 170-8381, Bio-Rad, Hercules, CA, United States) was used for image capture.

### 2.9 Statistical analyses

Statistical analyses were performed using the IBM Statistical Package for Social Sciences (SPSS) (version 27.0). The differences among means were analyzed using one-way analysis of variance (ANOVA) followed by the Games-Howell or Tukey’s *post hoc* tests.

## 3 Results

### 3.1 ppGpp competes with GTP and ATP for the binding to AtYchF1

**FIGURE 1:**
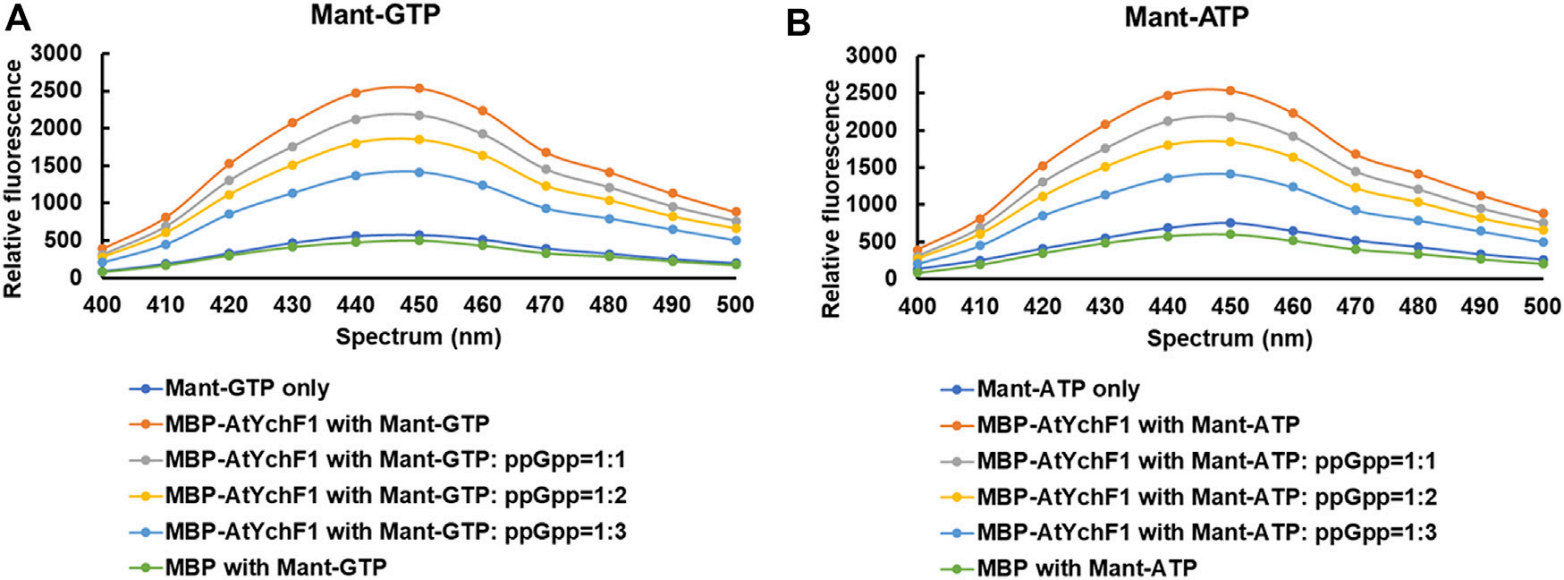
Nucleotide binding assays of AtYchF1. **(A)** The binding between MBP-AtYchF1 and GTP, and the ability of ppGpp to compete against Mant-GTP for binding with MBP-AtYchF1. **(B)** The binding between MBP-AtYchF1 and ATP, and the ability of ppGpp to compete against Mant-ATP for binding with MBP-AtYchF1. Relative fluorescence signals between 400-500 nm were recorded. MBP-only was included as a negative control.

The hydrolyses of GTP and ATP by AtYchF1 were previously reported (Cheung et al., 2013). To test the binding between AtYchF1 and ppGpp, the fusion protein MBP-AtYchF1 and the nucleotides Mant-GTP and Mant-ATP were used. Mant-GTP and Mant-ATP emit a fluorescent signal at 400-500 nm upon binding to a protein. When MBP-only was mixed with Mant-GTP or Mant-ATP, the intensity of the fluorescent signal was similar to the basal signal of Mant-GTP or Mant-ATP alone, showing that Mant-GTP or Mant-ATP did not bind to MBP (Figure 1). However, when MBP-AtYchF1 was added to Mant-GTP or Mant-ATP, the fluorescent signal increased above the baseline level, showing the binding between AtYchF1 and GTP or ATP. The ability of AtYchF1 to bind ppGpp was examined using Mant-GTP or Mant-ATP as the competing ligands. When ppGpp was added as a competitor to Mant-GTP or Mant-ATP for binding AtYchF1, the fluorescence intensity decreased with increasing concentrations of ppGpp. The result demonstrated that ppGpp competed well against Mant-GTP and Mant-ATP for the binding of MBP-AtYchF1 (Figure 1).

### 3.2 The hydrolysis of ppGpp by AtYchF1

**FIGURE 2:**
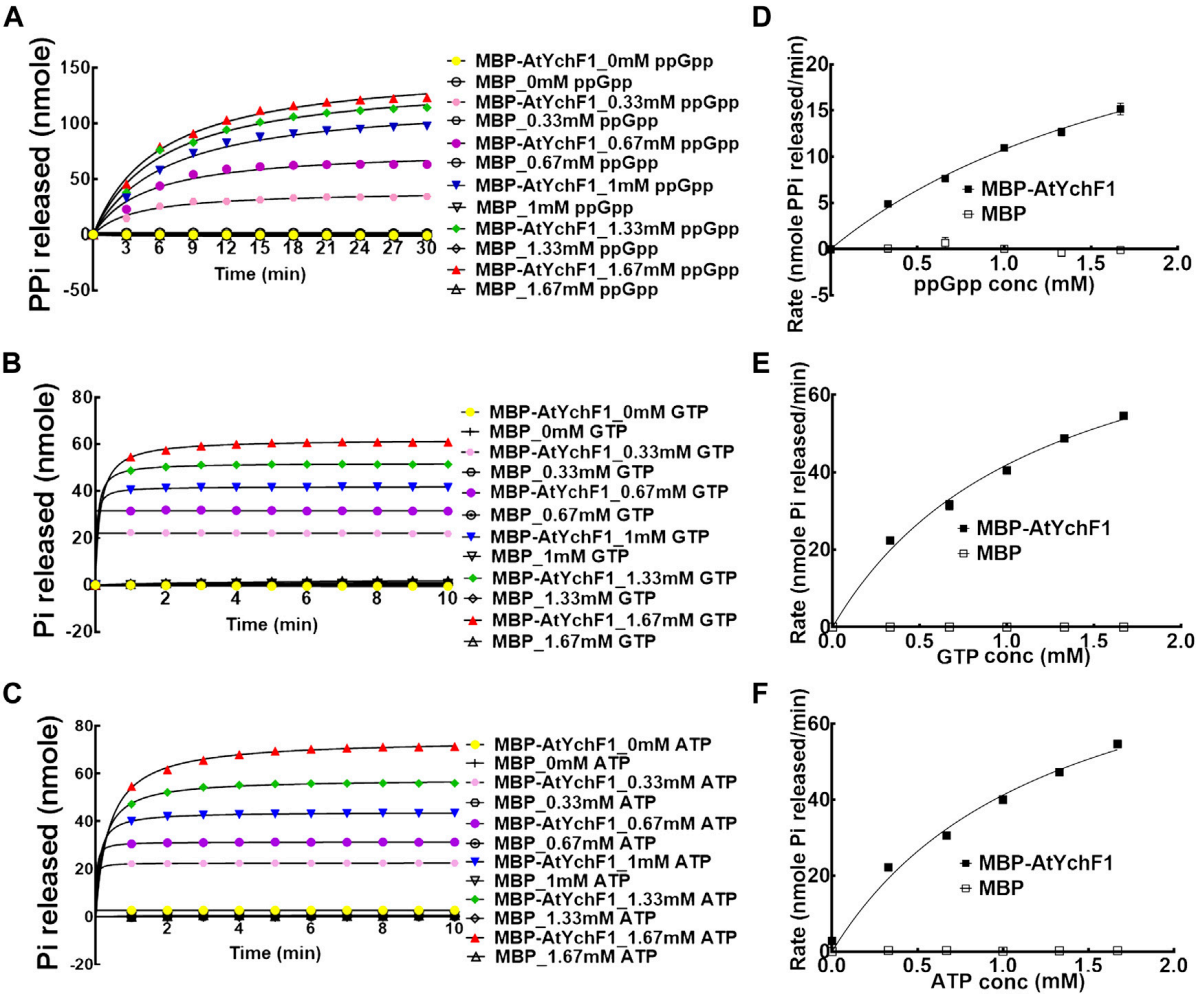
The hydrolytic activities of AtYchF1 on ppGpp, GTP, and ATP. The hydrolytic activity of MBP-AtYchF1 or MBP-only was tested with different concentrations of **(A)** ppGpp, **(B)** GTP and **(C)** ATP. **(D–F)** Michaelis-Menten plots of the rates at the initial log phase of ppGpp **(D)**, GTP **(E)** and ATP **(F)** by MBP-AtYchF1 or MBP-only.

The hydrolytic activities of AtYchF1 towards GTP, ATP and ppGpp were tested and compared. MBP-AtYchF1 and MBP-only were expressed and purified from *E. coli*. The release of inorganic phosphate (Pi) or pyrophosphate (PPi) was monitored with the use of EnzChek™ Phosphate Assay Kit or EnzChek™ Pyrophosphate Assay Kit, respectively, to indicate the hydrolytic activities. When ppGpp was mixed with MBP-AtYchF1, the release of PPi was detected (Figure 2A). The amount of PPi released increased with time and the amount of ppGpp added (Figure 2A). When GTP or ATP was mixed with MBP-AtYchF1, the release of Pi was detected (Figures 2B,C). The amount of Pi released increased with time and the amount of ppGpp added (Figures 2B,C). The kinetics of the hydrolytic activities were also tested using Michaelis-Menten plots (Figures 2D–F). Figure 2D shows the rates of PPi release at the logarithmic phase upon the hydrolysis by MBP-AtYchF1 with different concentrations of ppGpp while Figures 2E,F show the rates of Pi release at the logarithmic phase upon the hydrolysis by MBP-AtYchF1 with different concentrations of GTP (Figure 2E) and ATP (Figure 2F). In the hydrolysis assays, MBP-only was used as a negative control, and did not show any hydrolytic activity on ppGpp, GTP, or ATP. Using the Michaelis-Menten plots, the V_max_ and K_m_ values of AtYchF1 for ppGpp, GTP, and ATP were calculated by Prism 6 (GraphPad Software, Inc. Version 6.07) (Table 1). For ppGpp, the V_max_ and K_m_ values were 34.88 ± 2.974 nmole PPi min^−1^ and 2.235 ± 0.2943 mM, respectively (Table 1). The GTPase and ATPase activities of AtYchF1 were found to have similar V_max_ and K_m_ values. The V_max_ and K_m_ values of the GTPase activity were 92.4 ± 4.967 nmole Pi min^−1^ and 1.203 ± 0.1255 mM respectively, while those of the ATPase activity were 93.94 ± 7.142 nmole Pi min^−1^ and 1.278 ± 0.1838 mM respectively (Table 1). The results show that AtYchF1 has a lower affinity to ppGpp compared to GTP and ATP. The rate of hydrolysis of ppGpp by AtYchf1 is also lower compared to those of GTP and ATP.

**TABLE 1:** The K_m_ and V_max_ values of the hydrolysis of ppGpp, GTP, and ATP by AtYchF1.

	K_m_ (mM)	V_max_ (nmole/min)
ppGpp	2.235 ± 0.2943	34.88 ± 2.974
GTP	1.203 ± 0.1255	92.4 ± 4.967
ATP	1.278 ± 0.1838	93.94 ± 7.142

**FIGURE 3:**
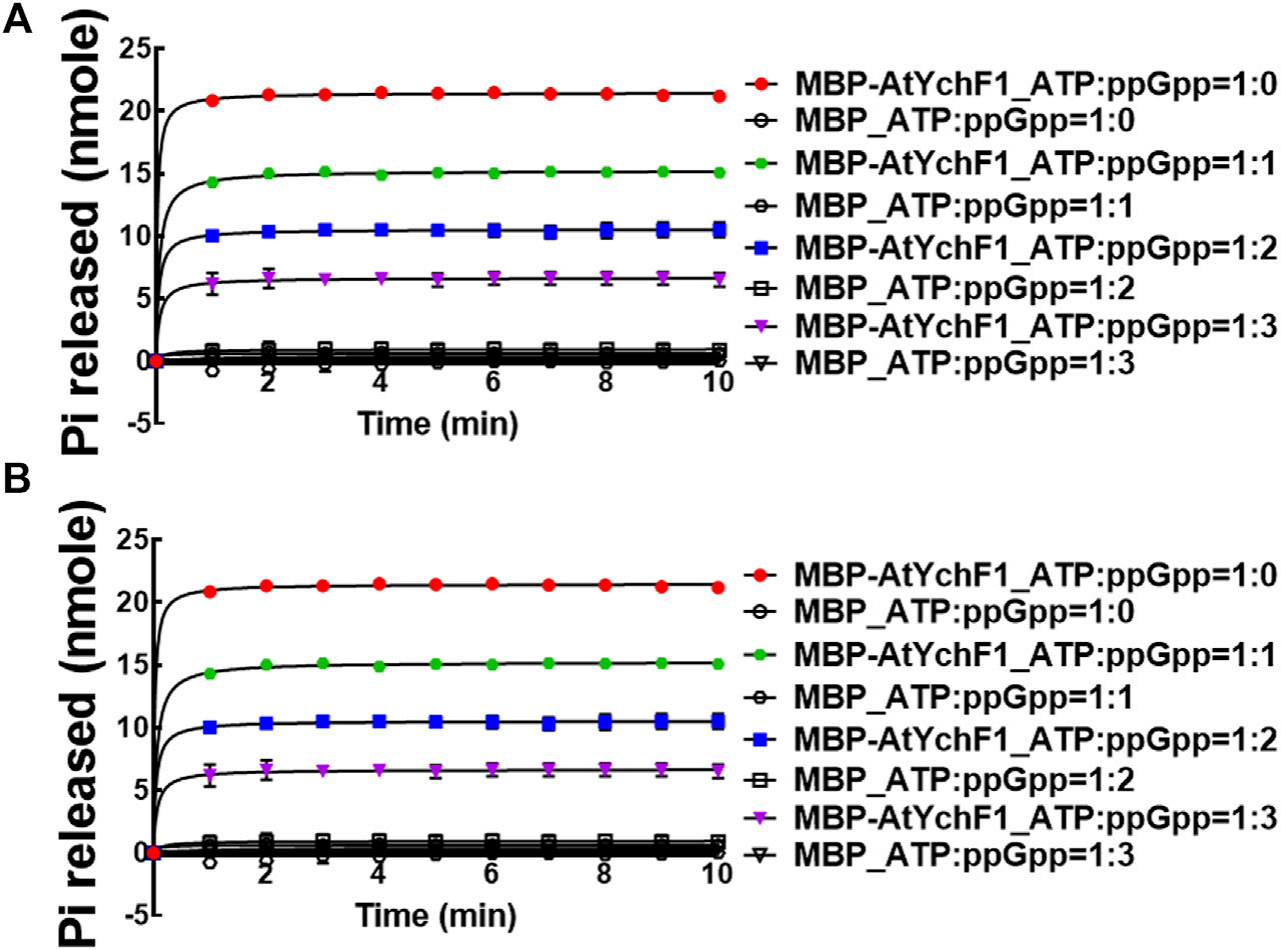
The competition of AtYchF1 hydrolytic activities on GTP and ATP by ppGpp. The hydrolytic activities of MBP-AtYchF1 on GTP **(A)** and ATP **(B)** were tested in the presence of increasing concentrations of ppGpp.

After determining the hydrolytic activities of AtYchF1 on ppGpp, GTP, and ATP, we then tested the competition between ppGpp and GTP or ATP for the AtYchF1 hydrolytic activity. In the competition assays, MBP-AtYchF1 or MBP-only at 0.3 mM was mixed with GTP or ATP at 0.33 mM, and ppGpp at 0, 0.33, 0.67 and 1 mM, to make up the GTP:ppGpp and ATP:ppGpp ratios at 1:0, 1:1, 1:2, and 1:3, respectively. The increase in ppGpp concentration led to a corresponding reduction in the GTPase and ATPase activities of MBP-AtYchF1 (Figure 3).

### 3.3 Co-crystalization of AtYchF1 and ppGpp

**FIGURE 4:**
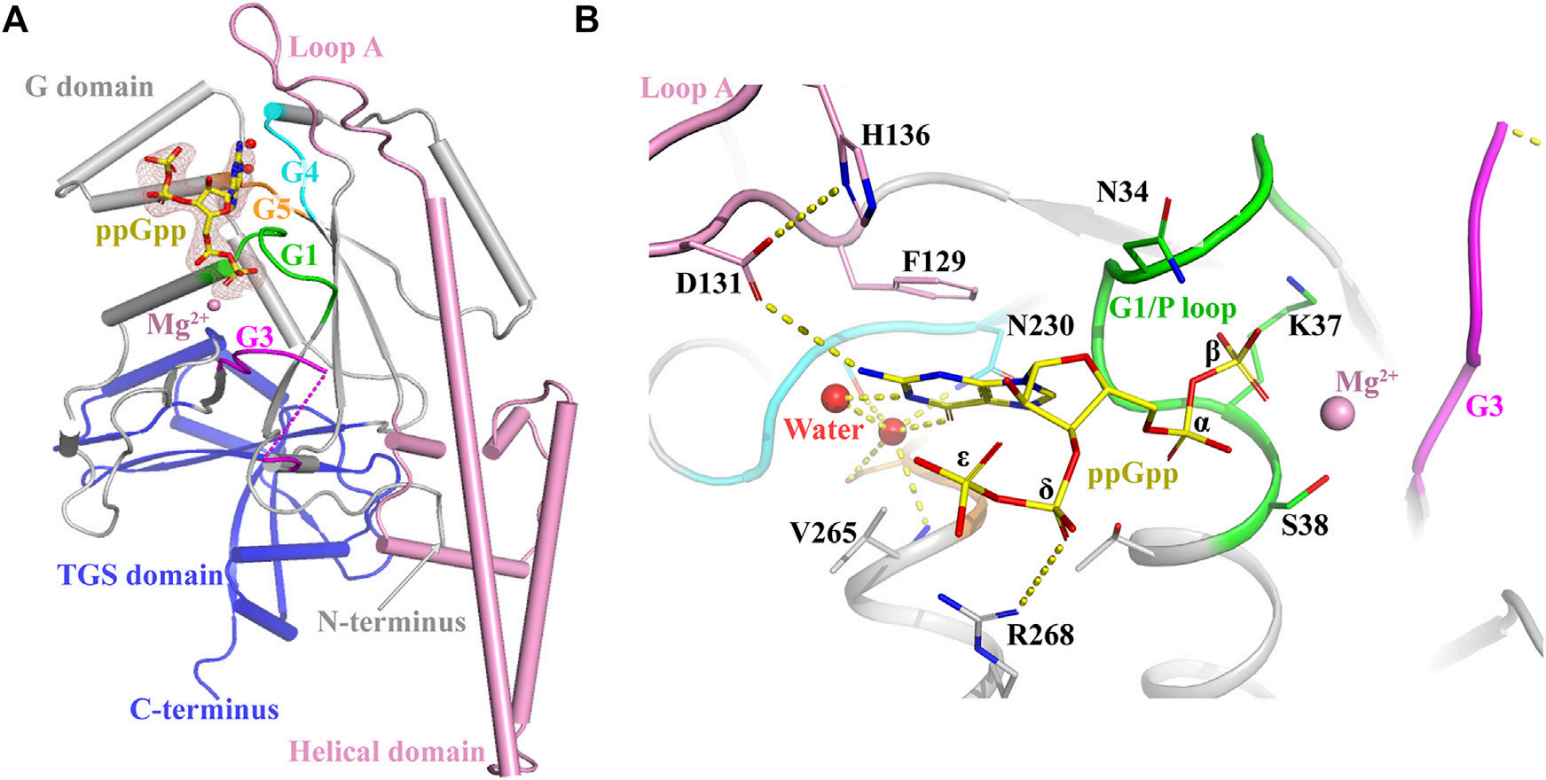
Crystal structure of the AtYchF1-ppGpp complex. **(A)** The overall structure of the AtYchF1-ppGpp complex. G domain, grey; helical domain, pink; TGS domain, blue. The G1, G3, G4, and G5 motifs of the G domain are colored green, magenta, cyan, and orange, respectively. *Fo*-*Fc* (3.0 σ), dark salmon mesh; ppGpp, yellow. Mg^2+^ ions and water molecules are shown as magenta and red spheres, respectively. **(B)** Detailed structure of the binding site on AtYchF1 for ppGpp. Hydrogen bonds or salt bridges are shown in dashed lines.

The conformational changes of OsYchF1 upon the binding to ATP or GTP were resolved by X-ray crystallography previously (Cheung et al., 2016). In this study, using X-ray crystallography, we resolved the conformational changes of AtYchF1 upon the binding to ppGpp. Since YchFs are highly conserved, we used the previously resolved structure of OsYchF1-AMPPNP or OsYchF1-GMPPNP as a reference to help analyze the structure of AtYchF1-ppGpp (Cheung et al., 2016). The structure of AtYchF1-ppGpp with Mg^2+^ was resolved using X-ray crystallography at 2.07Å. The ppGpp molecule could be unambiguously identified by and placed into the available densities (Figure 4). The statistics of the diffraction data collection and structure refinement are summarized in Supplementary Table S2. Overall, the structures of AtYchF1 and OsYchF1 are similar (Supplementary Figure S1A). Interestingly, the distance between the TGS and helical domains of the AtYchF1-ppGpp complex is 4.7 Å shorter than those in the OsYchF1-AMPPNP or OsYchF1-GMPPNP complex (Supplementary Figure S1A). This region is the putative site for double-stranded nucleic acid binding (Supplementary Figure S1B) (Teplyakov et al., 2003). For example, the TGS domain of OsYchF1 was identified to be the binding site of 26S RNA (Cheung et al., 2010). These differences in the distance between these two domains within the binding site may indicate the preference for different nucleotide substrates.

#### 3.3.1 The ppGpp binding site of AtYchF1-ppGpp co-crystal structure

In the structure of the AtYchF1-ppGpp complex, the ppGpp molecule was bound at the normal nucleotide-binding site of the G domain, as expected (Figure 4A). The guanine moiety of ppGpp was engaged in π-stacking interactions with the Loop A motif, sandwiched between the hydrophobic chains of Phe^129^ and Val^265^ (corresponding to Ala^265^ in OsYchF1). The O6 of the guanine ring was recognized by the well-described side-chain of Asn^230^ of the G4 element (Figure 4B). This mode of recognition is conserved between the OsYchF1-GMPPNP and AtYchF1-ppGpp structures. The α- and β-phosphates of ppGpp were coordinated by the G1 (Figure 4B) motif (P-loop), and an Mg^2+^ was coordinated by the β-phosphate and the side-chain of Ser^38^ in the G1 motif (Figure 4B). The δ- and ε-phosphates, which were covalently bound to the 3′-OH group of the ribose moiety, discriminated ppGpp from its GDP counterpart by pointing away from the active site. A salt bridge between the δ-phosphate and Arg^268^ was observed (Figure 4B). Due to the absence of a γ-phosphate, the binding of ppGpp was expected to be weaker than that of GTP or ATP (Supplementary Figure S2).

#### 3.3.2 The AtYchF1-ppGpp co-crystal structure suggests the conformational change of LoopA and G3/Switch II

Between the AtYchF1-ppGpp structure and the OsYchF1-AMPPNP/GMPPNP structures, there were some conformational differences in the functional motifs. The binding of ppGpp had a greater stabilizing effect on the conformation of Loop A (Arg^127^-Asp^142^), with His^136^ as the most significantly stabilized (Supplementary Figure S2). Loop A in the AtYchF1-ppGpp structure underwent nucleotide-dependent conformational changes compared with those in other YchF1-NTP structures (Supplementary Figures S1, S2). The highly conserved His^136^ residue in Loop A has been reported as critical for the ATPase activity in the YchF from *E. coli* (Rosler et al., 2015). However, in the AtYchF1-ppGpp structure, His^136^ was stabilized by Asp^131^ which had a hydrophilic interaction with the guanine moiety of ppGpp (Supplementary Figure S2). Thus, ppGpp might prevent His^136^ from executing nucleotide hydrolysis, and the mechanisms of the hydrolysis of ATP/GTP and ppGpp by AtYchF1 could then be different. Moreover, a notable structural difference also existed in the conformation of the G3/Switch II domain, which is known to be important for GAP proteins to activate nucleotide hydrolysis (Sprang, 1997) (Supplementary Figure S2). The Glu^104^ and Gln^106^ in the Switch II of AtYchF1 replaced the Ala^104^ and Glu^106^ of OsYchF1 (Supplementary Figure S3), respectively, so that the side-chain of Phe^112^ in α4 was oriented outwards (Supplementary Figure S2), and no longer formed a hydrophobic core with Ile^95^, Leu^98^, and Val^99^. Instead of forming an α3 helix as in OsYchF1, the Ile^95^-Val^99^ residues in AtYchF1 formed an extended loop structure, which stabilized the binding of ppGpp, by stabilizing water molecules coordinated with magnesium ions.

### 3.4 ppGpp inhibits the binding between AtYchF1 and 26S rRNA

**FIGURE 5:**
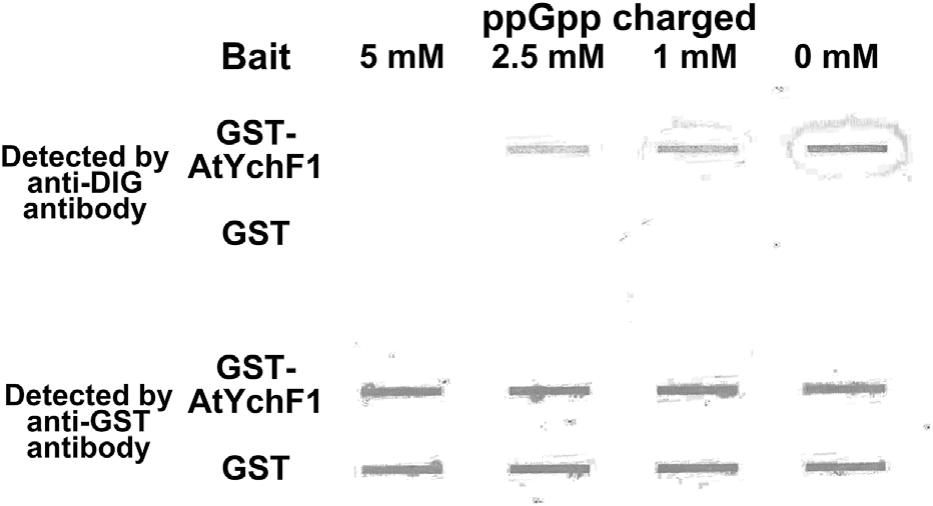
*In vitro* pull-down of digoxigenin (DIG)-labeled 26S rRNA by GST-AtYchF1 charged with different concentrations of ppGpp. *In vitro* pull-down of DIG-labeled 26S rRNA was done using magnetic bead-bound GST-AtYchF1 or GST-only charged with different concentrations of ppGpp (0, 1, 2.5 and 5 mM). The upper panel is the seating plan of pull-down products applied to the slot blot apparatus. GST-only charged with different concentrations of ppGpp were included as negative controls. The experiment was repeated for two times and similar results were obtained.

OsYchF1, which is a close homolog of AtYchF1, was reported to bind 26S rRNA (Cheung et al., 2010). In this study, inspired by the shorter distance between the TGS and helical domains of the AtYchF1-ppGpp complex compared to those in the OsYchF1-AMPPNP or OsYchF1-GMPPNP complex (Supplementary Figure S1A), we hypothesized that the binding of ppGpp to AtYchF1 may interfere with the binding between AtYchF1 and 26S rRNA. We first tested the binding between AtYchF1 and 26S rRNA by *in vitro* pull-down assay (Figure 5). In the *in vitro* pull-down assay, DIG-labeled 26S rRNA and GST-AtYchF1 were detected by anti-DIG antibodies and anti-GST antibodies, respectively (Figure 5). GST-only protein was employed as a negative control. Without being charged with ppGpp, the binding between GST-AtYchF1 and 26S rRNA was detected. However, when GST-AtYchF1 was charged with increasing concentrations of ppGpp, the binding between GST-AtYchF1 and 26S rRNA became progressively more inhibited (Figure 5).

### 3.5 AtGAP1 failed to activate the ppGpp hydrolyzing activity of AtYchF1

**FIGURE 6:**
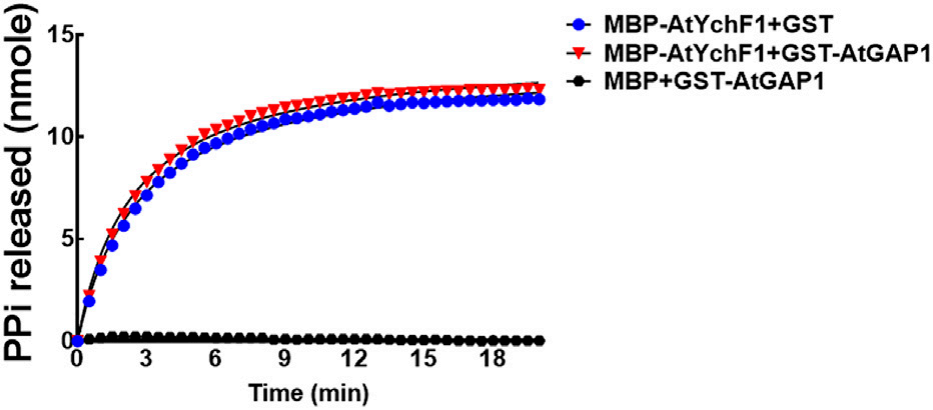
The ability of AtGAP1 to activate the hydrolysis of ppGpp by AtYchF1 was assessed by adding GST-AtGAP1 to the hydrolytic reactions. In the experiments, MBP-only was included as a negative control. The hydrolytic activity on ppGpp was recorded by detecting the release of pyrophosphate (PPi) during hydrolysis, using the EnzChek™ Pyrophosphate Assay Kit.

The ATPase and GTPase activities of OsYchF1 were previously shown to be activated by OsGAP1 (Cheung et al., 2013). In this study, however, we found that the ppGpp-hydrolyzing activity of AtYchF1 could not be further boosted by AtGAP1 (using 300 mM of ppGpp as substrate; Figure 6). The AtGAP1 protein used for the AtYchF1 activation assay was validated to be active (Supplementary Figure S4).

## 4 Discussion

In this report, we showed ppGpp as a novel ligand binding to YchF1 besides ATP and GTP which were shown previously (Cheung et al., 2016). The close homolog of AtYchF1, OsYchF1, was characterized as a cytosolic protein under normal condition (Cheung et al., 2010). Since AtYchF1 shows a high homology to OsYchF1 in terms of amino acid sequence and protein structure (Cheung et al., 2010), we speculate that AtYchF1 is also a cytosolic protein under normal condition. Such speculation is supported by the sub-cellular localization prediction by programs including TargetP 2.0 (Nielsen, 2021), Localizer 1.0.4 (CSIRO, 2015), WoLFPSORT (Nakai, 2004), Plant-mPLoc (Chou and Shen, 2010), and SignalP-5.0 (Nielsen, 2019) (Supplementary Table S3). The results suggested the cytosolic localization of AtYchF1. Therefore, the hydrolytic activities of AtYchF1 on nucleotides in the cytosol is supported.

In the binding competition experiment against GTP and ATP to the AtYchF1 protein, nearly three folds more ppGpp than either GTP or ATP was needed to reduce the fluorescent signal by 50% (Figure 1). The result demonstrated that the affinity of AtYchF1 to ppGpp was lower than that for ATP and GTP. The results are consistent with the ppGpp-AtYchF1 co-crystal structure suggesting the weaker binding between AtYchF1 and ppGpp, compared to GTP and ATP, due to the absence of a γ-phosphate (Supplementary Figure S2). Moreover, the hydrolytic activities of AtYchF1 on ppGpp was also lower than those on ATP and GTP, as reflected by the K_m_ and V_max_ values of the hydrolysis of the three nucleotides (Figure 2; Table 1). The hydrolytic activity of AtYchF1 on ATP or GTP was reduced by half when the concentration of ppGpp was double that of the ATP or GTP concentration (Figure 2; Table 1). Altogether, the results suggested that ppGpp could only compete with ATP and GTP as a substrate for the hydrolytic activity of AtYchF1 when the concentration of ppGpp was two to three folds higher than those of ATP and GTP. Although the existence of ppGpp in the cytosol of plant cells has remained unclear, the over-accumulation of ppGpp in the cytosol of Arabidopsis plants expressing the *Bacillus subtilis* ppGpp synthase gene (*yjbM*) was shown to inhibit plant growth (Ihara and Masuda, 2016). Hence, AtYchF1 could be a player in regulating the cytosolic ppGpp-mediated growth inhibition in plants.

The co-crystallization data suggest that the structures of OsYchF1 and AtYchF1 are similar (Supplementary Figure S1A). Using the structure of OsYchF1 as a reference, it was revealed that the distance between the TGS and helical domains of the AtYchF1-ppGpp complex is 4.7 Å shorter than those in the OsYchF1-AMPPNP or OsYchF1-GMPPNP complex (Supplementary Figure S1A). Such a phenomenon suggests that the binding of ppGpp may alter the double-stranded nucleic acid binding activity of AtYchF1. By charging the AtYchF1 protein with ppGpp, using *in vitro* pull-down assay, we showed that ppGpp inhibited the binding of AtYchF1 to 26S rRNA (Figure 5). YchF proteins have been proposed to be uncharacterized translation factors (Koller-Eichhorn et al., 2007; Gradia et al., 2009). The inhibitory effect of ppGpp on the binding between AtYchF1 and 26S rRNA hints at the possible role of ppGpp in regulating translation. In addition, the co-crystal structure revealed the conformational change in the G3/Switch II domain of AtYchF1 by ppGpp binding (Figure 2) and the consequent change in the ability to activate nucleotide hydrolysis by GAP proteins. The failure of AtGAP1 to activate the ppGpp-hydrolyzing activity of AtYchF1 was demonstrated (Figure 6). Altogether, the results suggest that AtYchF1 could regulate the level of ppGpp, which in turn regulates the functions of AtYchF1.

The binding between ppGpp and AtYchF1 shown in this report suggests the possibility of binding between ppGpp and YchF homologs in other species. ppGpp has been known as an alarmone for stress adaptations in bacteria and plants (Steinchen and Bange, 2016). The hydrolysis of ppGpp has long been known to be mediated by the hydrolase domains of RSH enzymes (Ronneau and Hallez, 2019). The results in this study hints at the possible regulation of the ppGpp level by YchF proteins in addition to RSH enzymes. This improved understanding on the regulation of ppGpp levels will enhance the study of ppGpp-regulated metabolic processes.

In this report, ppGpp was also demonstrated to compete with ATP, GTP, and 26S rRNA for the binding to AtYchF1 although the hydrolytic activity of AtYchF1 on ppGpp is lower than that on ATP or GTP (Figure 2). Therefore, ppGpp probably plays a negative role in regulating the functions of AtYchF1. Since AtYchF1 is a negative regulator of pathogen infection and salt stress (Cheung et al., 2010, 2013), ppGpp probably plays a positive role in stress adaptation. The inability of AtGAP1 to activate the ppGpp hydrolyzing activity of AtYchF1 (Figure 5) is in line with the positive role of AtGAP1 in regulating abiotic stress and biotic stress.

In conclusion, in this study, we demonstrated the binding and hydrolytic activity of AtYchF1 on ppGpp, which serves as an alarmone when under stress. Co-crystallization and *in vitro* pull-down results showed that ppGpp alters the structure of AtYchF1 and inhibits the binding between AtYchF1 and 26S rRNA. In addition, AtGAP1 failed to activate the hydrolytic activity of AtYchF1 on ppGpp. Since YchF proteins are conserved among different kingdoms of life, the results in this study suggest that YchF proteins, besides the well-known RHS enzymes, could also regulate ppGpp levels. In addition, the results suggest a possible role of ppGpp in regulating the functions of YchF proteins, which have been characterized as the regulators of both abiotic and biotic stresses in plant.

## Data Availability

The datasets presented in this study can be found in online repositories. The names of the repository/repositories and accession number(s) can be found below: http://www.wwpdb.org/, 7Y9I.
