# Supplementary material for: Co-crystalization reveals the interaction between AtYchF1 and ppGpp
Source: Front Mol Biosci. 2022 Nov 30;9:1061350. doi: 10.3389/fmolb.2022.1061350 (PMC9748339; doi:10.3389/fmolb.2022.1061350)
Supplement: Supplementary file 1 [file DataSheet1.docx]

Supplementary Material

**Table S1.** **Primers used in the experiments.**

| Construct | Description | Primer sequences |
| --- | --- | --- |
| pMAL-C2-*AtYchF1*,  pGex-4T-1-*AtYchF1* | Full-length amplification of *AtYchF1* cDNA with *EcoR*I and *Xho*I *for* subcloning into pMALC2 (*EcoR*I- and *Sal*I-digested) | Forward: 5’-AGTGAATTCTCTAGAATGCCTCCGAAAGCCAAAGCT-3’  Reverse: 5’-ACTCGAGTCATTTCTTCCCACCACC-3’ |
| PGex-4T-1-*AtGAP1* | Full-length amplification of *AtGAP1* cDNA with *EcoR*I and *Xho*I sites for subcloning into pGex-4T-1 (*EcoR*I- and *Sal*I-digested)) | Forward: 5’-AATCTAGAGAATTCATGACAACGGCGTGTCCG-3’  Reverse: 5’-AACTCGAGTCATAGACCCTTGGAGCC-3’ |
| pRESTA-HisSUMO-*AtYchF1* | Full-length amplification of *AtYchF1* cDNA with *Age*I and *Kpn*I sites for subcloning into pRSETA-HisSUMO | Forward: 5’-AAACCGGTGGAATGCCTCCGAAAGCCAAAG-3’  Reverse: 5’-AAGGTACCATCTCATTTCTTCCCACCACC-3’ |

**Table S2.** **Data collection and refinement statistics of the X-ray crystallography of AtYchF1-ppGpp.**

|  | AtYchF1-ppGpp (PDB: 7Y9I) |
| --- | --- |
| **Data collection** |  |
| Wavelength (Å) | 0.9792 |
| Space group | *P*2_1_2_1_2 |
| Cell dimensions |  |
| *a*, *b*, *c* (Å) | 72.46, 111.5, 52.33 |
| α, β, γ (°) | 90, 90, 90 |
| Resolution (Å)^a^ | 50.0-2.07 (2.12-2.07) |
| *R*_merge_(%) | 7.1 (139.4) |
| *I* / σ | 18.8 (2.2) |
| Completeness (%) | 99.9 (99.7) |
| Redundancy | 13.0 (13.3) |
| **Refinement** |  |
| Resolution (Å) | 50.0-2.07 (2.12-2.07) |
| No. of reflections | 25155 (1821) |
| *R*_work_ / *R*_free_ (%) | 20.6/23.6 |
| No. of atoms |  |
| Protein | 2785 |
| Ligands | 37 |
| Water | 61 |
| *B*-factors |  |
| Protein | 53.6 |
| Ligands | 61.5 |
| Water | 48.6 |
| R.m.s. deviations |  |
| Bond lengths (Å) | 0.006 |
| Bond angles (°) | 1.425 |
| Ramachandran Plot^b^ (%) | 92.5/7.5/0/0 |

^a^ Statistics for highest-resolution shell.

^b^ Residues in the most favored, additional allowed, generously allowed, and disallowed regions of the Ramachandran plot, respectively.

Table S3 Prediction of subcellular localization of AtYchF1 by TargetP 2.0, Localizer 1.0.4, WoLFPSORT, Plant-mPLoc, and SignalP-5.0. (NA: not applicable)

|  | **Mitochondria** | **Chloroplast** | **Nucleus** | | **Others** | | **Reference** | |
| --- | --- | --- | --- | --- | --- | --- | --- | --- |
| **TargetP 2.0** | 0.0011 | 0.0001 | NA | | 0.9975 | | (Nielsen, 2021) | |
| **Localizer 1.0.4** | 0% | 0% | 0% | | NA | | (CSIRO, 2015) | |
| **WoLFPSORT** | 0 | 0 | 0 | | cytoplasmic | | (Nakai, 2004) | |
| **Plant-mPLoc** | NA | NA | NA | | cytoplasmic | | (Chou; and Shen, 2010) | |
| **SignalP-5.0** | Signal peptide: 0.0015 | | | NA | | 0.9985 | (Nielsen, 2019) |  |

**
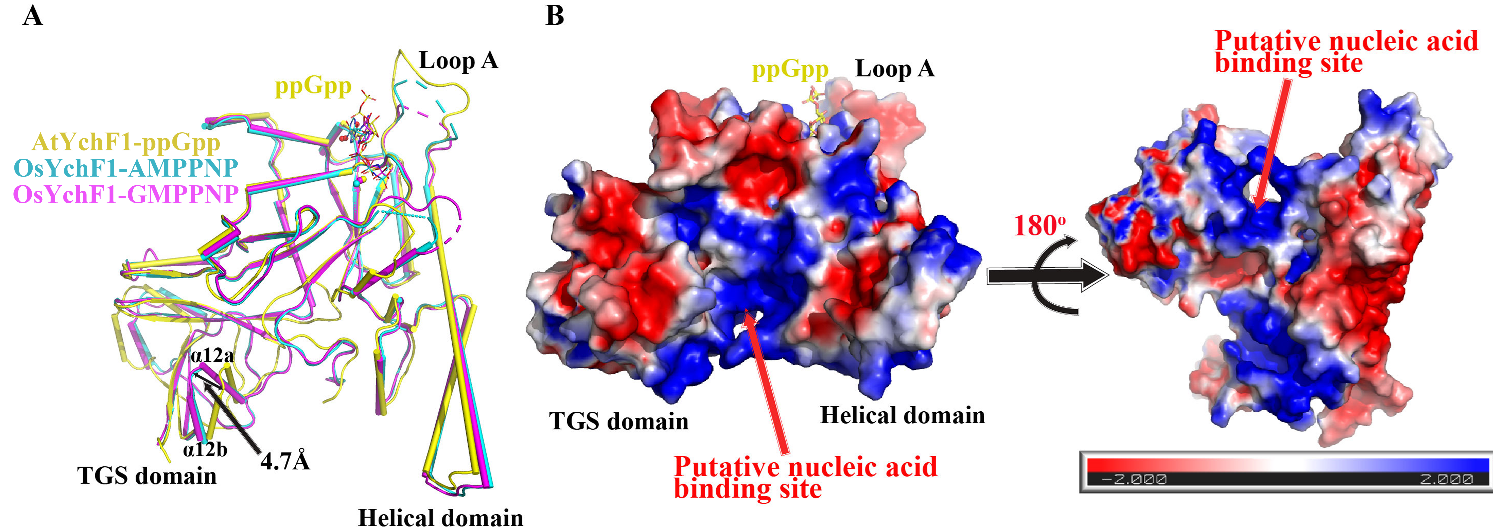
**

**Figure S1 Structural analyses of the AtYchF1-ppGpp complex.** (A) Structural alignment of the AtYchF1-ppGpp complex with the OsYchF1-AMPPNP and OsYchF1-GMPPNP complexes. (B) The distribution of surface charges of the AtYchF1-ppGpp complex.


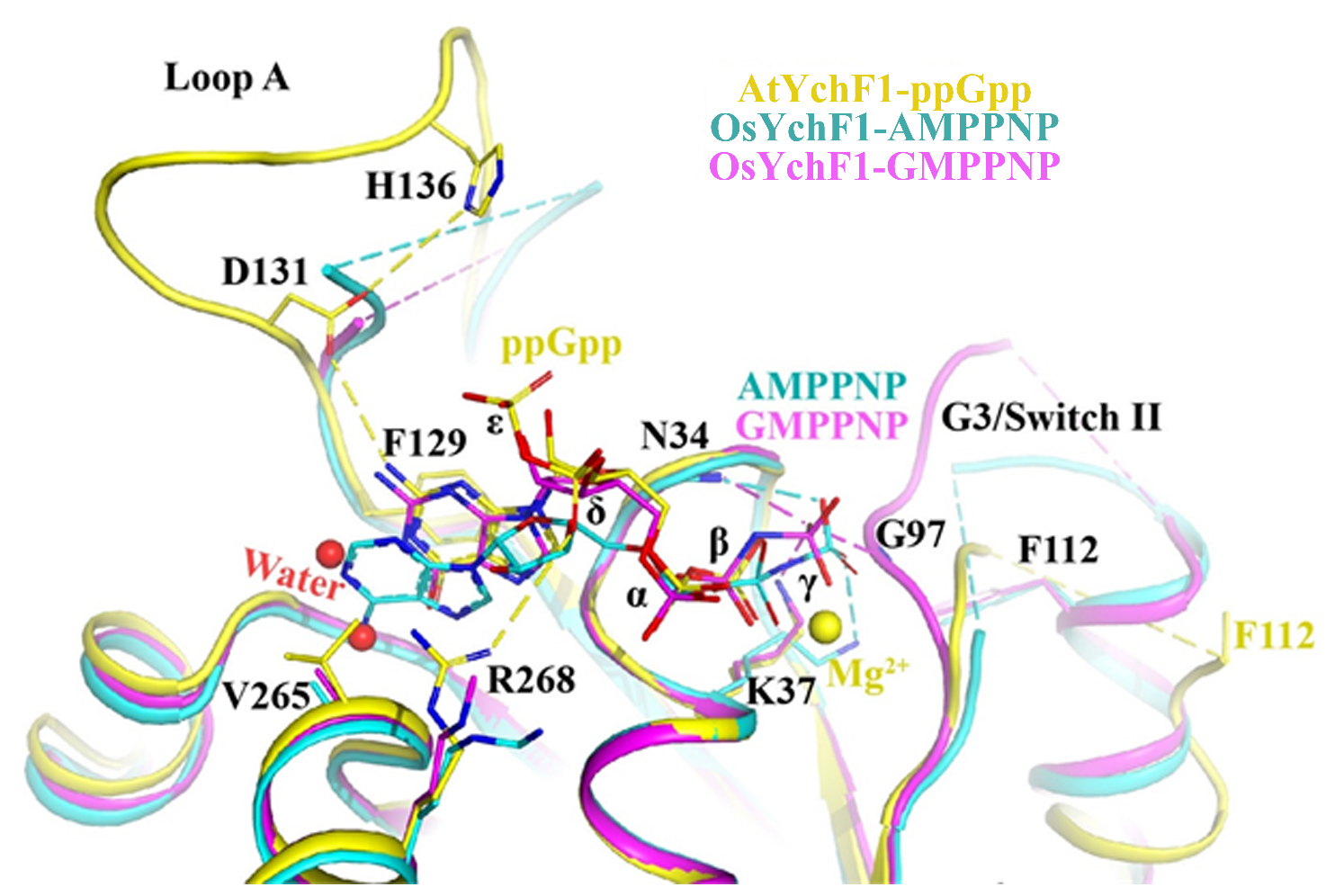


**Figure S2 Structural comparisons between the ppGpp-binding region of the AtYchF1-ppGpp complex and the ligand binding sites of the OsYchF1-AMPPNP and OsYchF1-GMPPNP complexes. The interacting residues between γ-phosphate of ATP or GTP and OsYchF1 and the hydrogen bonds were labelled.**


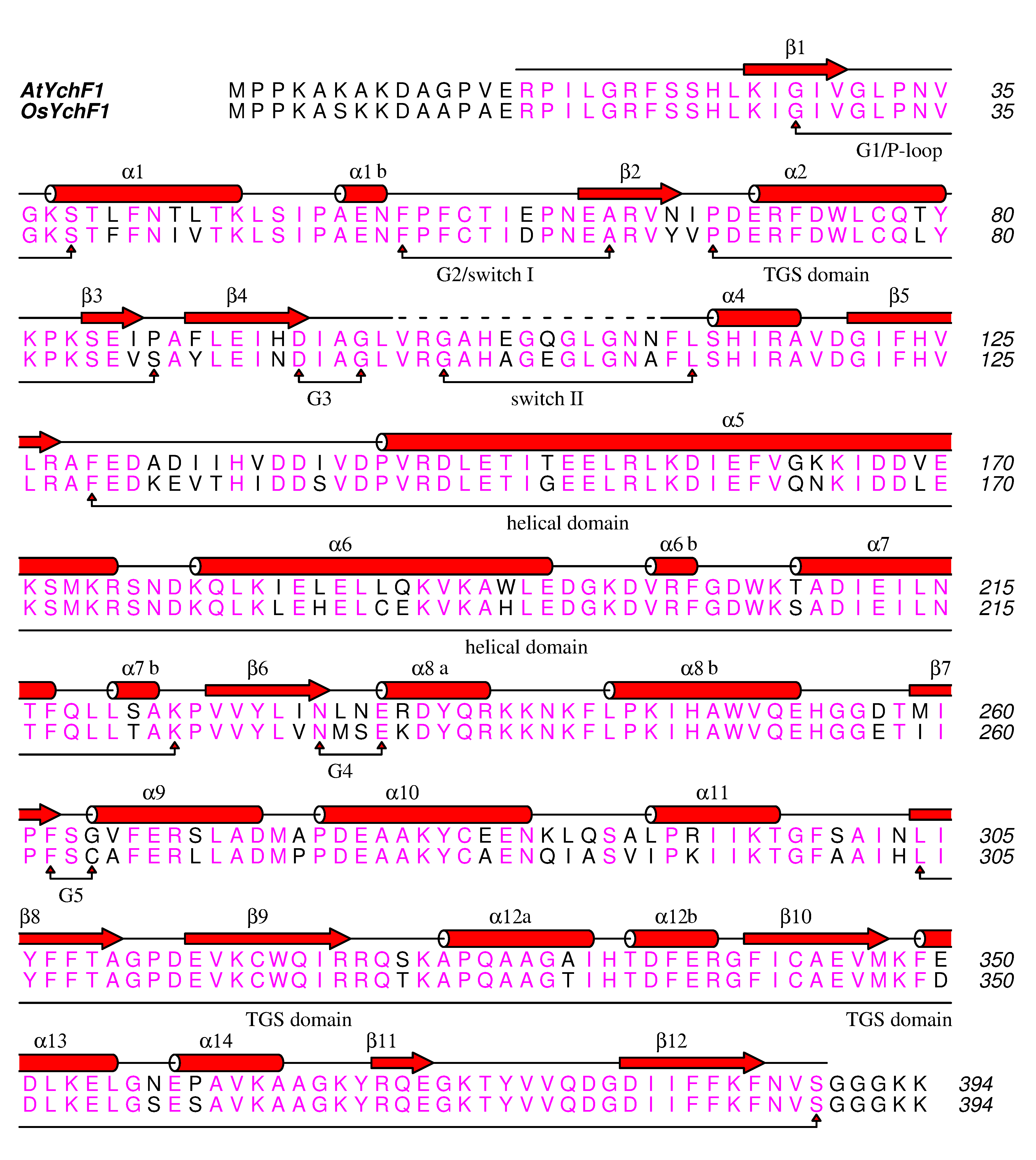


**Figure S3 Structure-based sequence alignments of OsYchF1 and AtYchF1.** Secondary structural elements are represented according to the structure of AtYchF1-ppGpp. Cylinders and arrows represent α-helices and β-sheets, respectively. Invariable residues are highlighted. The secondary structural elements are numbered according to the convention used in HiYchF (Teplyakov et al., 2003) and hOLA1 (Koller-Eichhorn et al., 2007).


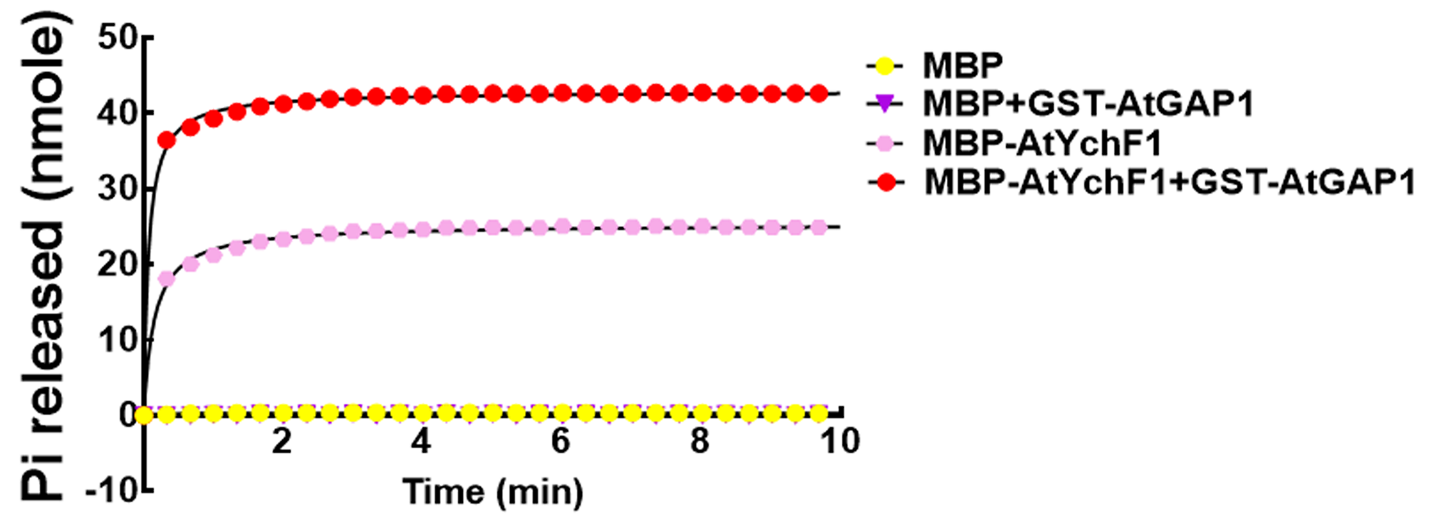


**Figure S4 Activity test of the same batch of AtGAP1 protein used for ppGpp hydrolysis activation assay.** The activity of the AtGAP1 protein was shown by its capacity to activate the GTPase activity of AtYchF1. MBP-only was included as a negative control. The hydrolytic activities on GTP were recorded by detecting the release of inorganic phosphate during hydrolysis using the EnzChek™ Phosphate Assay Kit.

References:

Chou;, K.-C., and Shen, H.-B. (2010). Plant-mPLoc. Available at: http://www.csbio.sjtu.edu.cn/bioinf/plant-multi/# [Accessed November 1, 2022].

CSIRO (2015). LOCALIZER: subcellular localization prediction of plant and effector proteins in the plant cell. Available at: https://localizer.csiro.au/ [Accessed November 1, 2022].

Koller-Eichhorn, R., Marquardt, T., Gail, R., Wittinghofer, A., Kostrewa, D., Kutay, U., et al. (2007). Human OLA1 defines an ATPase subfamily in the Obg family of GTP-binding proteins. *J. Biol. Chem.* 282, 19928–19937.

Nakai, K. (2004). WoLF PSORT Protein Subcellular Localization Prediction. Available at: https://wolfpsort.hgc.jp/ [Accessed November 1, 2022].

Nielsen, H. (2019). SignalP-5.0. Available at: https://services.healthtech.dtu.dk/service.php?SignalP-5.0 [Accessed November 1, 2022].

Nielsen, H. (2021). TargetP - 2.0. Available at: https://services.healthtech.dtu.dk/service.php?TargetP-2.0 [Accessed November 1, 2022].

Teplyakov, A., Obmolova, G., Chu, S. Y., Toedt, J., Eisenstein, E., Howard, A. J., et al. (2003). Crystal structure of the YchF protein reveals binding sites for GTP and nucleic acid. *J. Bacteriol.* 185, 4031–4037.
